# Diffuse Large B-Cell Lymphoma (DLBCL) in an Aged Raccoon (*Procyon lotor*)

**DOI:** 10.3390/vetsci9110611

**Published:** 2022-11-04

**Authors:** Giulia D’Annunzio, Luca Gelmini, Giuseppe Sarli, Andrea Luppi, Francesca Gobbo, Patrizia Bassi, Gianluca Rugna

**Affiliations:** 1 Istituto Zooprofilattico Sperimentale della Lombardia e dell’Emilia Romagna, Via A. Bianchi 9, 25124 Brescia, Italy; 2 Dipartimento di Scienze Mediche Veterinarie, University of Bologna, Via Tolara di Sopra 50, 40064 Ozzano dell’Emilia, Italy

**Keywords:** cancer, DLBCL, lymphoma, neoplasia, raccoon, wildlife

## Abstract

**Simple Summary:**

The monitoring of wildlife disease patterns is important because wild species represent the human–environment interface, making them useful sentinels for both human and animal health. We aimed to classify the lymphoid neoplasm detected during necropsy based on the topography, cell type, and immunophenotype of neoplastic lymphocytes in a captive raccoon. There is substantial evidence showing the histopathological similarities among many tumors, even in unconventional species, which may represent possible alterative models worthy of investigation. In our study, the successful use of immunohistochemistry with clinically relevant human and canine biomarkers allowed us to diagnose multicentric diffuse large B cell lymphoma in raccoons.

**Abstract:**

A 15-years-old, captive, female raccoon (*Procyon lotor*) was necropsied after a one-week history of apathy and self-isolation. Gross changes consisted of the severe enlargement of the mesenteric lymph node; hepatosplenomegaly with multifocal to coalescing, white tan nodules in the spleen and liver,; and pale kidneys. Histologically, neoplastic CD79α-positive lymphocytes effaced the mesenteric lymph node and multifocally infiltrated the spleen, liver, and kidneys, and focally infiltrated the heart. Based on pathological and immunohistochemical findings, as well as the canine-adapted World Health Organization (WHO) diagnostic criteria, a diagnosis of diffuse large B-cell lymphoma (DLBCL) was made.

## 1. Introduction

Cancer-related deaths are common in mammals and occur across various taxa, although it is very challenging to detect cancer in wild populations [1]. Indeed, cancer-related mortality risk studies are mainly conducted on captive animals with a longer average lifespan than free-ranging wildlife [1,2].

In raccoons (*Procyon lotor*), neoplasms are rarely documented but the occurrence of cancer in raccoon populations might be underestimated due to the difficulty in estimating morbidity and mortality rates related to spontaneous diseases in wildlife [3]. Recent reports of neoplasm in raccoons include descriptions of raccoon polyomavirus-associated brain tumors [4,5,6], and a few offers concerning epithelial neoplasms [7,8,9]. Regarding round cell tumors, there are only two other earlier cases of lymphosarcoma in raccoons, both of which lack immunophenotype characterization [10,11]. Instead, in coati (*Nasua nasua*), another member of the Procyonidae family, the occurrence of two different entities of T-cell lymphoma has been documented: epitheliotropic cutaneous lymphoma (ECL) and intestinal T-cell lymphoma [12,13]. A study of the immunophenotype of lymphocyte cells in tumors of wild animals was also performed in other cases, such as in the raccoon dog (*Nyctereutes procyonoides*) [14].

Here, according to the canine-adapted World Health Organization (WHO) diagnostic criteria [15], we described a case of multicentric diffuse large B-cell lymphoma (DLBCL) in an aged, captive, female raccoon.

## 2. Case Presentation

The carcass of a 15-year-old female raccoon (*Procyon lotor*), reared in captivity, was submitted to the Istituto Zooprofilattico Sperimentale della Lombardia e dell’Emilia Romagna for necropsy. The subject had been apathic and lethargic, and tended to isolate itself during the week before death.

**Figure 1:**
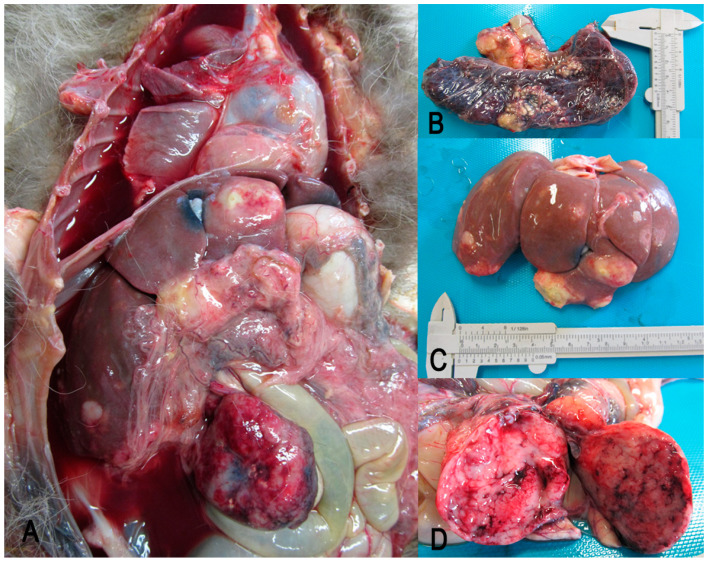
Gross findings. (**A**) Overall macroscopic features of abdominal and thoracic viscera at necropsy. (**B**) Spleen is expanded by white tan nodules, multifocal to coalescing. (**C**) Liver is increased in volume and is characterized by multiple raised white nodules. (**D**) Enlarged mesenteric lymph with a tan light-pinkish cut surface.

On gross examination, the raccoon was in poor body condition with a severe reduction in subcutaneous and visceral fat. The examination of the abdominal cavity revealed hepato-splenomegaly with both tissues expanded by multiple, often coalescing, white tan nodules, variable in size from 0.5 to 2 cm in diameter, bulging on the cut surface (Figure 1A–C). The mesenteric lymph node was markedly enlarged (Figure 1D) and the surface of the parenchyma was light-pinkish and tan after being cut. Bilaterally, the kidneys were pale and characterized by cortical discolored areas, and were multifocal to coalescing. Other gross findings comprised pulmonary edema, a moderate amount of serosanguinous abdominal and thoracic effusions, as well as a pale and dilated heart with no other lesions visible to the naked eye. The intestine was poorly preserved, and the other lymph nodes examined were normal. 

**Figure 2:**
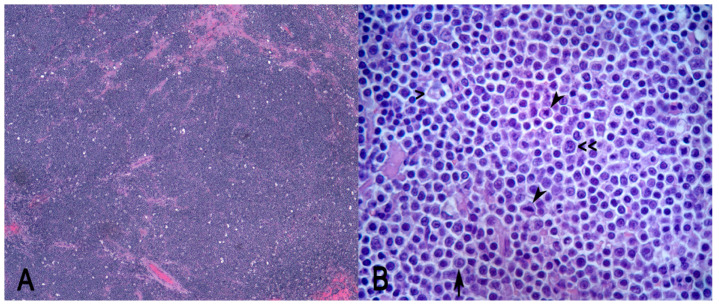
Diffuse large B-cell lymphoma (DLBCL) in raccoons. (**A**) A typical “starry sky” appearance resulting from sheets of round neoplastic cells mixed with scattered histiocytes containing phagocytosed cellular debris. (**B**) Tangible body macrophages containing phagocytosed debris at high magnification (>); nucleolar arrangements of the neoplastic cells are both centroblastic (<<) and immunoblastic (arrow); mitoses are numerous (arrowheads). Hematoxylin–eosin (HE). (**A**) Magnification 40×; (**B**) magnification 630×.

Samples of the mesenteric lymph node, small and large intestine, spleen, liver, kidney, heart, and lung were processed for histopathology. A histopathological examination of tissue sections routinely stained with hematoxylin–eosin (HE) stain revealed monomorphic lymphomatous infiltration affecting the mesenteric lymph node, spleen, liver, kidneys, and heart. Few other microscopic changes were detected, including multiple foci of pneumoconiosis in the lung and bilateral, chronic, multifocal, and tubulointerstitial nephritis in the kidneys. Intestinal sections were diffusely autolytic. The mesenteric lymph node appeared effaced after being non-demarcated and unencapsulated, and densely cellular neoplasm composed by sheets of round cells were supported by scant fibrovascular stroma. Neoplastic cells were characterized by distinct cell borders, a high N/C ratio, and a scant amount of eosinophilic cytoplasm. Nuclei were 2× the size of red blood cells and were hyperchromatic with coarsely stippled chromatin. The nucleolar arrangement was variable; it was characterized by multiple peripherally located nucleoli (centroblast-like) or a single and central nucleolus (immunoblast-like) (Figure 2B). Anisocytosis and anisokariosis were moderate and mitosis was 22 in 2.37 mm^2^. Neoplastic cells were mixed with numerous tangible body macrophages which conferred a “starry-sky” microscopic appearance to the neoplasm (Figure 2A). Intratumorally areas of necrosis and hemorrhage, which were multifocal to coalescing, were detected.

The Immunophenotype of neoplastic cells was detect by immunohistochemical (IHC) labeling for T and B cell antigens (CD3 and CD79α) (Table 1).

**Figure 3:**
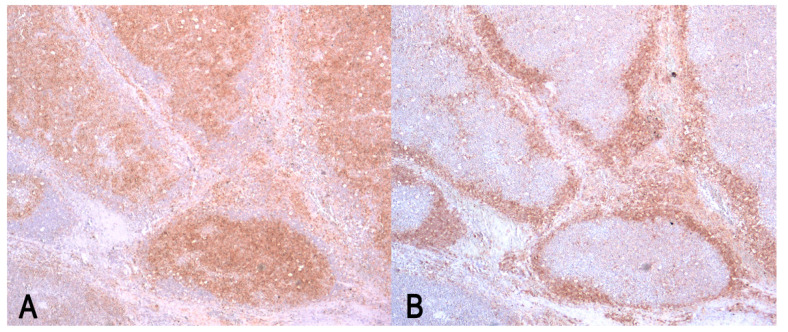
Diffuse large B-cell lymphoma (DLBCL) in mesenteric lymph node. (**A**) Plasma membrane CD79α-positive stain of neoplastic B cells. Immunolabeling with anti-CD79α; (**B**) CD3-negative stain of neoplastic cells; the neoplasia destroys the normal nodal architectures and the expansion of neoplastic cells in paracortex peripherally displaces the CD3-positive non-neoplastic T-cells. Immunolabeling with anti-CD3. (**A**,**B**) Magnification 40×.

The neoplastic cells were strongly positive for CD79α and were negative for CD3 (Figure 3).

**Figure 4:**
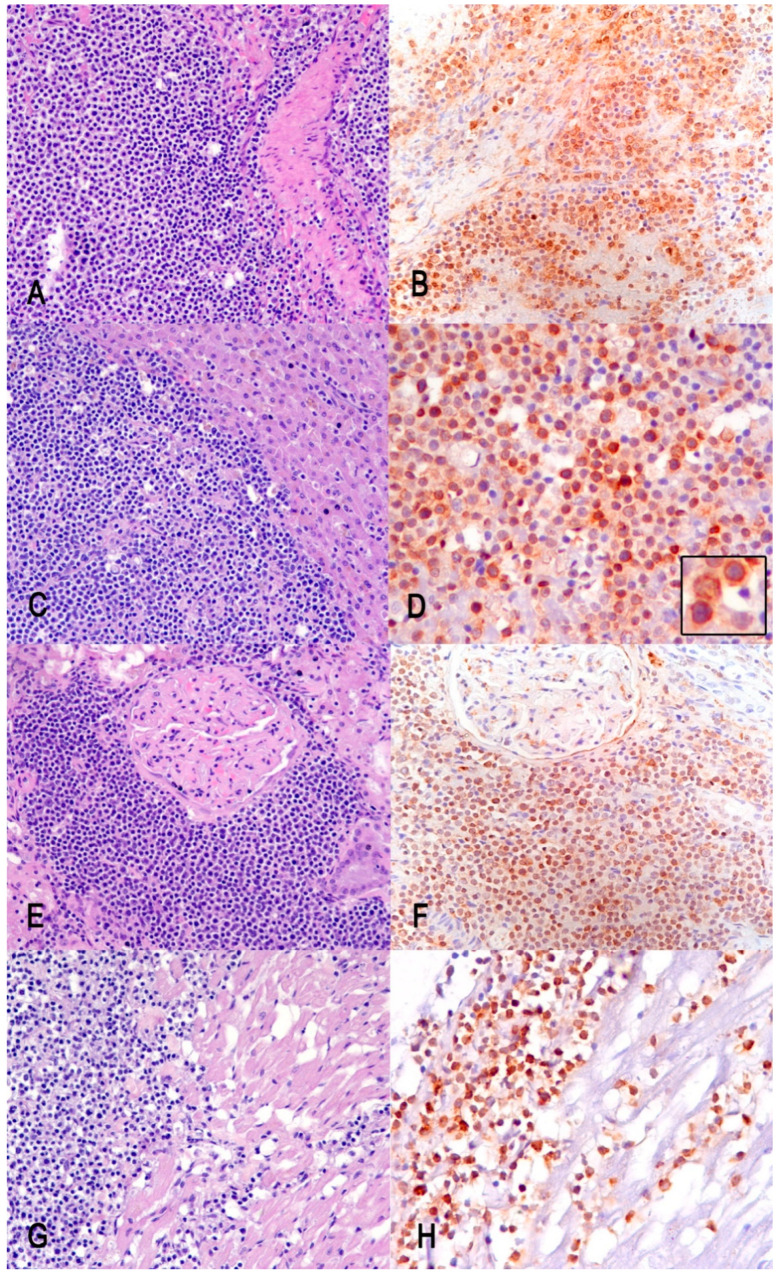
Multiorgan infiltration of neoplastic B cells. Hematoxylin–eosin (HE) and CD79α-positive immunohistochemistry stain (IHC), respectively, of the spleen (**A**,**B**), the liver (**C**,**D**), the kidney (**E**,**F**), and the heart (**G**,**H**). Insert in D: details of the membranous CD79α-positive stain. (**A**–**C**,**E**–**G**) Magnification 200×; (**D**,**H**) magnification 400×.

The diffuse infiltration of CD79α-positive neoplastic cells was detected in the spleen, liver, and kidneys, and was focally detected in the heart (Figure 4).

## 3. Discussion

According to the canine-adapted WHO diagnostic criteria [15], we performed the diagnosis of multicentric diffuse large B-cell lymphoma (DLBCL). We aimed to apply the canine-adapted WHO guidelines to classify the observed neoplasms, according to the topography, cell type, and immunophenotype of neoplastic lymphocytes. 

The WHO diagnostic classification is commonly used in domestic animals. However, there is evidence of histopathological similarities in many tumors, even in unconventional species, which may represent possible alterative models worthy of investigation [16].

Regarding the growth pattern and tumor architecture, in our case, the diffuse arrangement of sheets of neoplastic cells effacing all the mesenteric lymph node allowed us to differentiate a diffuse form from a nodular one.

The tumor cell morphology was consistent with the defining features of diffuse large B-cell lymphoma [15]; this is because in DLBCL, based on nuclear sizes, the neoplastic cells were large (nuclei ≥ 2 red cells in diameter) and were characterized by a scant cytoplasm. As in many cases of DLBCL, the cells had both types of nucleolar arrangement: the one referred to as immunoblastic (i.e., one central nucleolus) and the other one with multiple nucleoli located at the nuclear periphery (i.e., the DLBCL centroblastic type).

The amount of mitotic figures was quite high and the typical “starry sky” appearance was observed at low magnification, resulting from the intra-tumoral scattered histiocytes with phagocytosed cellular debris.

As in the canine DLBCL (or, in this case, lymphoma in raccoon), the cells were neoplastic B cell, negative to CD3 and strongly positive to CD79α. Although both monoclonal antibodies used in the IHC analysis are not specific for the examined species, they were chosen on the basis of the previous study of Heinrich et al. [17], which demonstrated the cross-reactivity of these antibodies with the lymphocyte populations in the lymphoid tissues of the raccoon. 

In our case, based on the anatomic location, the multicentric form of DLBCL lymphoma was hypothesized. Unfortunately, the intestine was markedly autolytic and we could not exclude the intestinal origin of lymphoma, since the mesenteric lymph node was the most severely involved tissue. In cats, alimentary lymphoma is the most common gastrointestinal neoplasm; malignant lymphocytes infiltrate the intestine, with or without involvement of other abdominal organs or bone marrow, but without lesions in the thorax or the peripheral sites [18]. In dogs, intestinal lymphoma usually spreads to mesenteric lymph nodes and to the liver. In the case presented here, the presence of monomorphic CD79α-positive infiltrate in the heart leads us to consider the multicentric form of lymphoma.

## 4. Conclusions

Diffuse large B-cell lymphoma is the most commonly detected mature lymphoid malignancy in humans and dogs, sharing a similar clinical presentation and similar morphological features to the neoplastic cells [19].

Similar aspects of cancer exist in a number of different species. The monitoring of wildlife patterns of diseases is important because wild species represent the human–environment interface and they are useful sentinels for both human and animal health.

In racoons, reports of lymphoid neoplasm are limited, making it difficult to estimate the incidence or to make conclusions about the most common entity of lymphoma in this species.

Nevertheless, in this study, we report the successful use of immunohistochemistry to make a proper diagnosis in a spontaneous tumor in a wild species, using a clinically relevant biomarker, both in humans and in dogs. This helps to expand the knowledge of cancer in wildlife.

## Figures and Tables

**Table 1:** Immunohistochemistry, materials, and methods.

Marker	Type and Clone	Supplier	Dilution/Incubation	Ag Retrieval	Esternal Positive Control
CD79α	Mouse monoclonal anti-human antibodies (clone HM57)	Santa Cruz Biotechnology Inc., Dallas, TX, USA	1:400/ON 4 °C	10′ EDTA pH8 MW: 750 W	Canine lymph node
CD3	Mouse monoclonal anti-human antibodies (clone CD3-12)	Leukocyte Antigen Biology Laboratory, Peter F. Moore, University of California, Davis, CA, USA	1:10/ON 4 °C	10′ Citrate pH6 MW: 750 W 0.05% at 37°	Canine lymph node

## Data Availability

Not applicable.
